# Plant sizes mediate mowing‐induced changes in nutrient stoichiometry and allocation of a perennial grass in semi‐arid grassland

**DOI:** 10.1002/ece3.3866

**Published:** 2018-02-16

**Authors:** Zhiying Liu, Taogetao Baoyin, Juan Sun, Hugjiltu Minggagud, Xiliang Li

**Affiliations:** ^1^ Key Laboratory of Grassland Ecology School of Ecology and Environment Inner Mongolia University Hohhot China; ^2^ College of Animal Science and Technology Qingdao Agricultural University Qingdao China; ^3^ Key Laboratory of Grassland Ecology and Restoration of Ministry of Agriculture National Forage Improvement Center Institute of Grassland Research Chinese Academy of Agricultural Sciences Hohhot China

**Keywords:** ecological stoichiometry, functional traits, mowing, phenotypic plasticity

## Abstract

While mowing‐induced changes in plant traits and their effects on ecosystem functioning in semi‐arid grassland are well studied, the relations between plant size and nutrient strategies are largely unknown. Mowing may drive the shifts of plant nutrient limitation and allocation. Here, we evaluated the changes in nutrient stoichiometry and allocation with variations in sizes of *Leymus chinensis*, the dominant plant species in Inner Mongolia grassland, to various mowing frequencies in a 17‐yr controlled experiment. Affected by mowing, the concentrations of nitrogen (N), phosphorus (P), and carbon (C) in leaves and stems were significantly increased, negatively correlating with plant sizes. Moreover, we found significant trade‐offs between the concentrations and accumulation of N, P, and C in plant tissues. The N:P ratios of *L. chinensis* aboveground biomass, linearly correlating with plant size, significantly decreased with increased mowing frequencies. The ratios of C:N and C:P of *L. chinensis* individuals were positively correlated with plant size, showing an exponential pattern. With increased mowing frequencies, *L. chinensis* size was correlated with the allocation ratios of leaves to stems of N, P, and C by the tendencies of negative parabola, positive, and negative linear. The results of structure equation modeling showed that the N, P, and C allocations were co‐regulated by biomass allocation and nutrient concentration ratios of leaves to stems. In summary, we found a significant decoupling effect between plant traits and nutrient strategies along mowing frequencies. Our results reveal a mechanism for how long‐term mowing‐induced changes in concentrations, accumulations, ecological stoichiometry, and allocations of key elements are mediated by the variations in plant sizes of perennial rhizome grass.

## 1 INTRODUCTION

In grassland ecosystems, the roles of plant nutrient strategies in plant‐soil feedback, biogeochemical cycling, and land nutrient management are increasingly recognized (Fry et al., 2017; Yuan & Chen, 2009). At the individual plant level, the concentrations, stoichiometry, and resorption efficiency of nitrogen (N), phosphorus (P), and carbon (C) have important influences on plant growth, reproduction, and competitive ability, particularly in nutrient‐poor ecosystems (Vergutz, Manzoni, Porporato, Novais, & Jackson, 2012). Some previous studies provided evidences for scale dependence of plant nutrient strategy to changes of environmental factors (Li, Hou, et al., 2016). For example, some empirical studies reported divergent responses in grassland plant stoichiometry to N addition and mowing at species and community levels (Han, Sistla, Zhang, Lu, & Han, 2014). Moreover, a recent analysis from a standardized nutrient addition experiment conducted at 42 nutrient network sites in eight countries showed that grassland productivity is limited by multiple nutrients (Fay et al., 2015). Accordingly, an understanding of plant nutrient strategies at organ level is of great theoretical interest; it reveals the underlying mechanisms of biogeochemical cycling and is therefore of importance for improving the productivity and quality of agro‐grassland systems.

Traditionally, mowing is an important aspect of land‐use management in arid and semi‐arid natural grasslands and is frequently performed by local herdsmen in Inner Mongolia (Baoyin, Li, Bao, Minggagud, & Zhong, 2014). Experimentally, defoliation, that is, the removal of plant photosynthetic shoot tissue, is a key mechanism by which large herbivores affect ecosystem functioning of grasslands and can be simulated by mowing (Chen et al., 2014). Mowing affects plant growth directly by removing and damaging photosynthetic tissues and indirectly by affecting biologically regulated processes in the soil through its influence on soil organisms (Carey, Beman, Eviner, Malmstrom, & Hart, 2015; Delaney, 2012). It is possible that mowing negatively affects plant nutrient acquisition and growth by decreasing root mass, but this effect is species specific (Cheplick, 1998; Han, Luo, & Du, 2007; Zhao, Chen, & Lin, 2008). The effects of mowing‐induced changes on nutrient strategies within individual plants are still poorly understood.

Plant nutrient strategies, ecological stoichiometry and nutrient allocation, for example, are always size dependent (Ågren, 2008). In general, small plants were relatively enriched in N and relatively depleted in P compared to larger plants (Méndez & Karlsson, 2005). In Inner Mongolia grassland, Bai et al. (2012) reported that plant stoichiometric responses to grazing ranged from large in the meadow steppe to small in the typical steppe to generally insignificant in the desert steppe. However, the question of how mowing‐induced changes in plant nutrient strategies are mediated by plant phenotypic traits of this species has not been experimentally tested in semi‐arid grasslands.

In addition, typical steppes dominated by *Leymus chinensis*, a native and perennial rhizomatous grass, cover the largest area in the eastern Eurasian temperate grassland along the border to China (Li, Liu, et al., 2015). This study is a continuation of work previously presented (Li, Hou, et al., 2016). Our previous study demonstrated that the allometric scaling of different phenotypic traits in *L. chinensis* leaves and stems varies with different mowing intensities, which is likely to be an ecological strategy used by *L. chinensis* in adapting to abiotic disturbances (Li, Hou, et al., 2016). Here, this study used a long‐term mowing intensity experiment to evaluate the effects of mowing on the concentrations, accumulations, ecological stoichiometry, and allocations of key elements in *L. chinensis*. We address the novel question of the relations of phenotypic traits (e.g., plant height and leaf size) and nutrient strategies of *L. chinensis*. We addressed three main questions: (1) How does mowing influence the relationships between *L. chinensis* size and C, N, and P concentrations in leaves and stems? (2) How does mowing intensity affect the relationship between *L. chinensis* C:N:P stoichiometry and morphological plasticity? (3) How does mowing intensity affect C, N, and P allocation patterns in *L. chinensis* with varying plant sizes?

## 2 MATERIALS AND METHODS

### 2.1 Study site

Our field experimental site was located at the Inner Mongolia Grassland Ecosystem Research Station (IMGERS, 43°38′N, 116°42′E, 1,200 a.s.l.) of the Chinese Academy of Sciences in the Xilinhot, Inner Mongolia Autonomous Region, P.R. China. The semi‐arid continental climate of this area is characterized by a mean (1998–2013) precipitation of 253.45 mm and a mean temperature of 13.77°C during grassland growth season (April to August). Maximum precipitation usually falls during the growing season (June, July, and August), coinciding with the highest temperatures in this period. The coefficient of variation for precipitation is over 20% because of the inter‐annual variability (Li, Hou, et al., 2016). The major soil types of this area are calcic chestnut and calcic chernozem. Before this experiment began, *L. chinensis* (a perennial rhizomatous grass) and *Stipa grandis* (a perennial bunchgrass) typically dominated the grassland communities of our study area (Bai et al., 2004; Li, Liu, et al., 2015).

### 2.2 Experimental design and sampling

The establishment of experimental area has been described in detail by Li, Hou, et al. (2016) and Li, Liu, et al. (2016). Here we expounded the experimental design briefly. The experimental treatments were contained by a long‐term continuous mowing exclusion, and three frequencies of mowing in a randomized block design, which was established nearly two decades ago (beginning in 1998). All the permanent plots were 10 m × 10 m in size. The information of the treatments was (1) CK, no mowing; (2) M_1/2_, mowing once every 2 years; (3) M_1/1_, mowing once a year; and (4) M_2/1_, mowing twice a year. The mowing was conducted each year on 16 August (or 1–3 days later depending on the weather) for the M_1/2_ and M_1/1_ treatments and on 15 June and 15 September for the M_2/1_ treatment. The lawn mower was used to cut the grass to a 6‐cm stubble height (Li, Hou, et al., 2016; Li, Liu, et al., 2016). Three replicate plots in each treatment were used in this study. In each plot of all of the mowing treatments, five 1 × 1 m^2^ quadrats were randomly selected in 2013. In all plots of the different mowing regimes, temporary markers were set up at each sampling point prior to clipping before the growing season in early April 2013, avoiding the abnormal disturbances of sampling quadrats in mowing plots. The field sampling was carried out on 15–20 August 2013 corresponding to the time of annual peak‐standing biomass of *L. chinensis*. Three *L. chinensis* individuals were randomly selected for measurement in each of the quadrats. All of the leaves and stems from each individual were sampled and measured according to their phytomer position from the base to the apex (Coelho, Valério, & Monteiro, 2009; Yang, Auerswald, Bai, Wittmer, & Schnyder, 2011).

### 2.3 Measurements

Using the method described by (Pérez‐Harguindeguy et al., 2013), the measurement of phenotypic traits of these individuals, including leaf length (LL*)*, leaf width (LW), leaf number (LN), stem length (SL), stem diameter (SD), and plant height (PH), has been described in our previous study (Li, Hou, et al., 2016; Li, Liu, et al., 2016). Leave areas of *L. chinensis* were scanned using a digital scanner and then were measured using the software of Photoshop. Subsequently, leaves and stems were packed in separate paper bags and were oven‐dried at 65°C for 48 hr and weighed for leaf biomass (LBM), stem biomass (SBM), and aboveground biomass (ABM).

The samples of *L. chinensis* leaves and stems were smashed using a mechanical micromill and passed through a 40‐mesh sieve. The total concentration of C in leaves and stems was determined using the H_2_SO_4_–K_2_Cr_2_O_7_ oxidation method (Bennett, Judd, & Adams, 2003). Total concentration of N was analyzed using the Alpkem autoanalyzer (Kjektec System 1026 Distilling Unit, Sweden), and total concentrations of P was determined colorimetrically at 880 nm after reaction with molybdenum blue. All stoichiometric ratios of *L. chinensis* leaves and stems C:N:P were calculated as mass ratios (Lü, Lü, Zhou, Han, & Han, 2012).

### 2.4 Statistical analysis

In our statistical analysis, the measured values of all the leaves from the base to the apex in one individual were averaged to represent the leaf phenotypic traits. Then, the mean values of each of the leaf or stem phenotypic traits were calculated by the measurements taken from three *L. chinensis* individuals in a single quadrat. Primarily, the method of principal components analysis (PCA) was performed to determine the relationships among the phenotypic traits and the effects of different mowing frequencies on these traits (Fort et al., 2015; Li, Hou, et al., 2016). For this analysis, all variables were normalized because they had different units. The importance of a phenotypic trait of *L. chinensis* for a given component was indicated by its relative loading on the component. The significance of these loadings was tested using Pearson's correlation test for all traits of *L. chinensis* individuals (Bagousse‐Pinguet, Bello, Vandewalle, Leps, & Sykes, 2014). Secondly, one‐way ANOVAs with Duncan's multiple‐range tests were performed across all variables in plant traits response to mowing intensity (Chen et al., 2016). The degree of response of *L. chinensis* traits to mowing was analyzed using the plasticity index (PI) given by the following equation (Moreno & Bertiller, 2015):

(1) $$\text{PI}=\frac{\text{FU}-\text{FM}}{\text{FU}}\times 100\%,$$

where FU represents the phenotypic traits of plants in without mowing treatment and FM represents the phenotypic traits of plants subjected to different mowing frequencies.

The allocation ratios of leaves to stems in N, P, and C were calculated by allocation ratio (AR) given by the following equation:

(2) $$\text{AR}=\frac{\text{LE}}{\text{SE}},$$

where LE and SE represent the accumulation amount of N, P, and C in *L. chinensis* leaves and stems, respectively.

In addition, the direct or indirect pathways of mowing frequencies on individual *L. chinensis* N, P, and C allocation patterns were analyzed by structural equation modeling (SEM) method (Byrne, 2013). The SEMs were developed based on our hypothesized relationships between variables and tests of preliminary models. The final SEMs were applied to each of the *L. chinensis* nutrient‐related indices (i.e., leaf to stem ratios of nutrient concentrations and biomass allocation). The utility of each nutrient index within the SEM was compared based on a number of measures, including the power of the particular model to explain the variation in *L. chinensis* N, P, and C allocation (*r* ^2^), measures of model significance and fit (χ^2^), and the significance of the functional trait variables within the model (Li, Hou, et al., 2016). The SEM was performed using the IBM SPSS AMOS 18 software packages.

## 3 RESULTS

### 3.1 Phenotypic plasticity in responds to mowing

**Figure 1:**
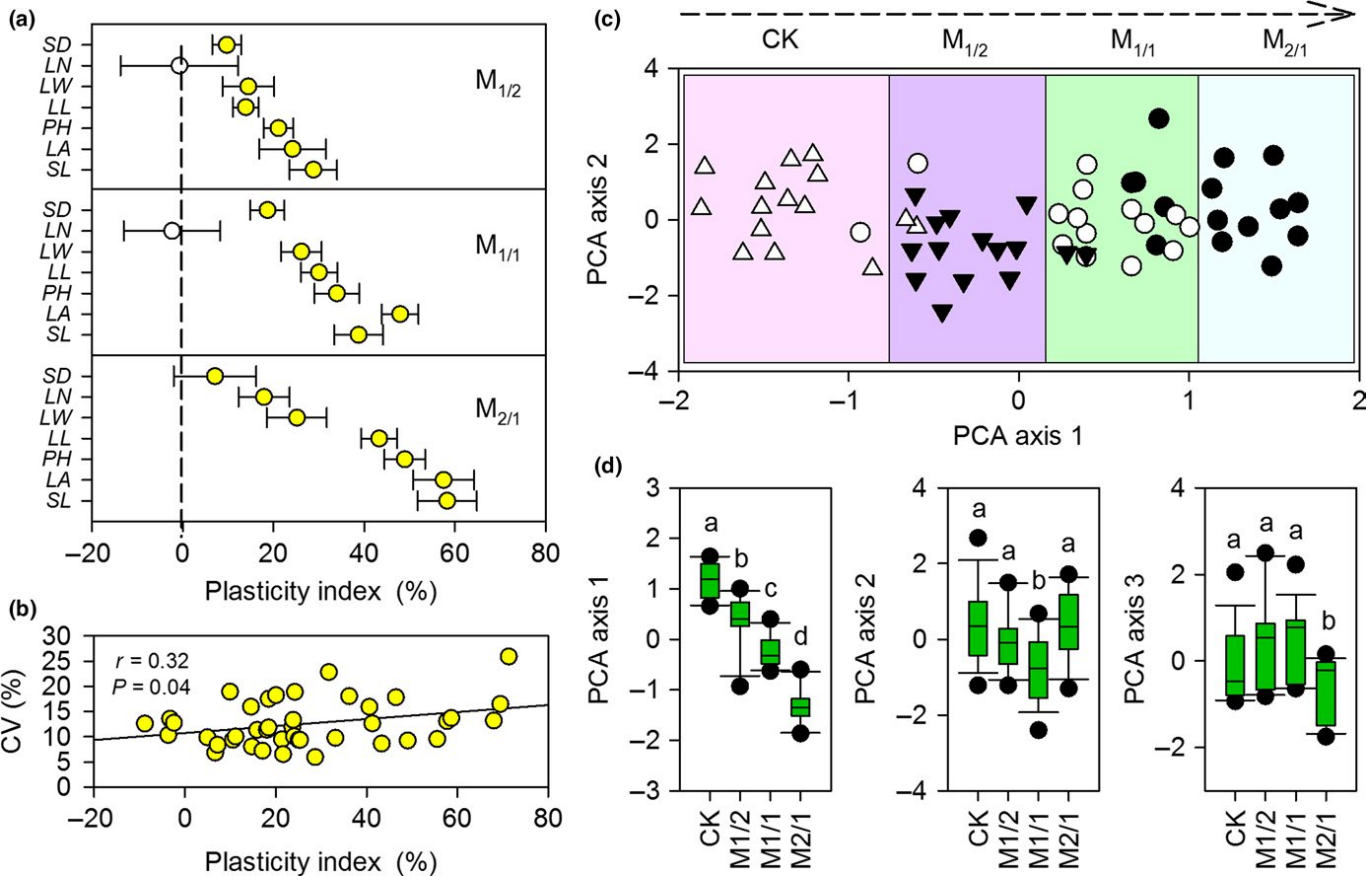
Effects of different clipping frequencies on the phenotypic plasticity of *Leymus chinensis*. (a) Plasticity indexes (mean ± standard deviation) of *L. chinensis* functional traits according to clipping intensity. (b) Relationship between the plasticity index and coefficient of variation (CV) of *L. chinensis* functional traits. (c) PCA bi‐plot of *L. chinensis* functional traits for the four treatments explained by the first (PCA axis 1) and second (PCA axis 2) principal axes. (d) Box plots illustrate the score distribution of *L. chinensis* functional traits from the four experimental communities along the three principal axes. Solid circles and empty circles in Figure 1a represent a significant effect of clipping and no significant effect of clipping on *L. chinensis* functional traits, respectively. Symbols in Figure 1c: ▵, CK (no clipping); ▾, M_1/2_ (herbage harvested once every second year); ○, M_1/1_ (herbage harvested once every year); ●, M_2/1_ (herbage harvested twice every year). Different letters in Figure 1d indicate significant difference (*p *< .05). LN *,* Leaf number; LL *,* leaf length; LW *,* leaf width; LA *,* leaf area; SL *,* stem length; SD *,* stem diameter; PH *,* plant height

Mowing had significantly negative effects on majority of the leaf and stem phenotypic traits (*p *<* *.05). The exception to this is the increasing of LN per *L. chinensis* individual under light mowing disturbance (Figure 1a). Based on our results, leaf and stem phenotypic traits can be classified into sensitive (i.e., PH and SL) and insensitive traits (i.e., LN and SD) according to the level of variation (from about 0.00% to 60%) in PI (Figure 1a). Mowing significantly increased the variability of plant functional traits (*p *<* *.05). The CV values of plant traits were significantly correlated with PI (*p *<* *.05, Figure 1b). Based on the PCA results, we found that the first PCA axis, which explained 74.04% of the total variance, mainly represented *L. chinensis* plant size as estimated by height (Figures 1c and S1). The second PCA axis, which accounted for 16.71% of the total variance, was strongly associated with stem and leaf width (Figure 1c, Table S1). Mowing significantly decreased the loading score of plant size along PCA axis 1, PCA axis 2, and PCA axis 3 (Figure 1d).

### 3.2 Trade‐offs between plant size and nutrient concentrations

**Figure 2:**
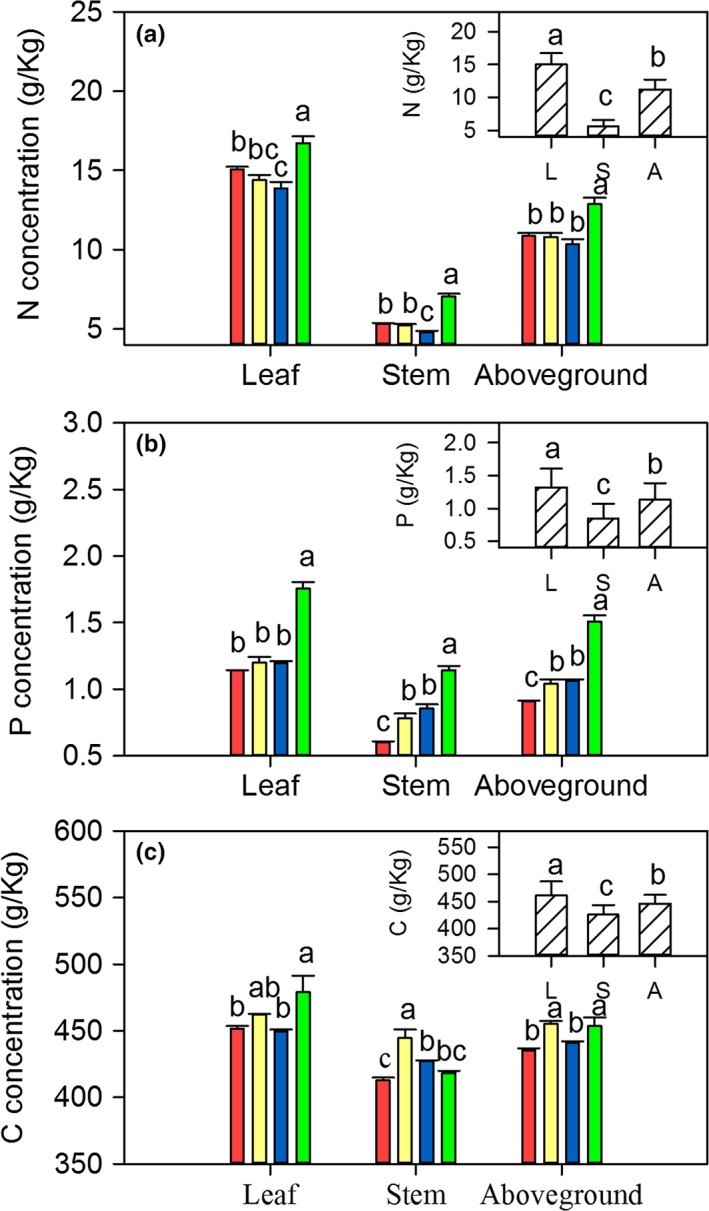
Effects of long‐term mowing frequencies (large figures) and sampling position (small figures) on N (a), P (b), and C (c) concentrations of *Leymus chinensis* individuals. The different colors in the large figures represent the four mowing frequencies (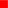, Control; 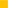, 1/2‐cut; 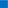, 1‐cut, 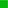, 2/1‐cut). The upper cases of L, S, and A in the small figures represent sampling positions (L, leaf; S, stem; A, aboveground, i.e., leaf + stem). Data represent mean ± *SE* values; different letters above the error bars indicate significant difference (*p* < .05)

**Figure 3:**
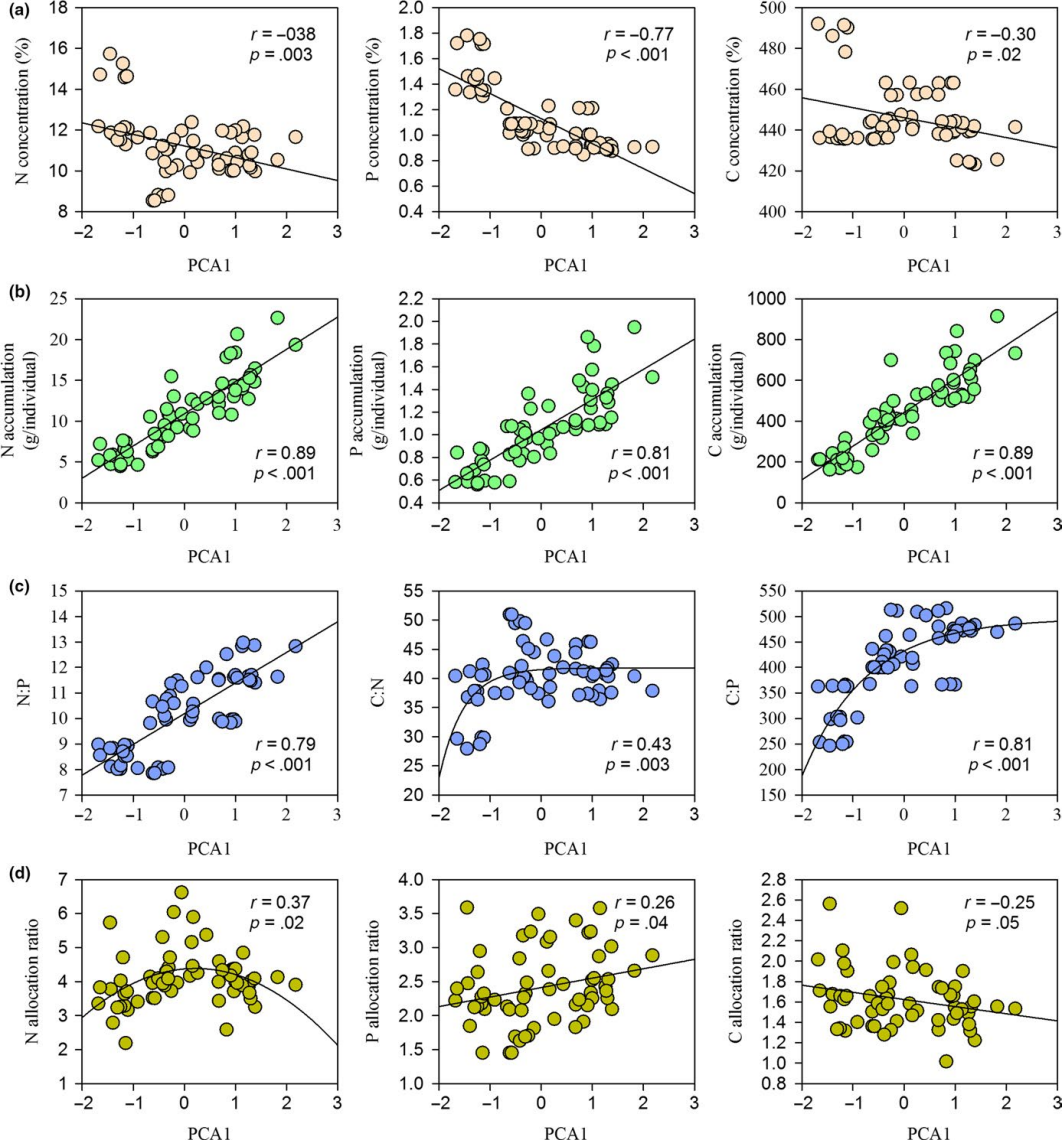
Nutrient concentrations (a), nutrient accumulation (b), ecological stoichiometry (c), and nutrient allocation (d) of *Leymus chinensis* individuals in response to the variations of plant size along with mowing frequencies. The relationships of PCA1 with C:N and C:P were fitted by the equation of Exponential Rise to Maximum, the relationship between PCA1 and N allocation ratio was fitted by quadratic equation while the other relations were fitted by linear regression equation

Mowing significantly increased the concentrations of N, P, and C in leaves and stems (*p *<* *.05, Figure 2), albeit with differences between the three elements. The N concentrations in treatment M_2/1_ but M_1/1_, M_1/2_ had significant difference with control treatment (Figure 2a). In contrast, increasing mowing intensity gradually increased P concentrations (Figure 2b). In comparison with N and P, the relation of C concentrations and mowing frequencies was fluctuant (Figure 2c). In relative terms, the nutrient concentrations in leaves were significantly higher than in stems, especially in terms of N (*p *<* *.05, Figure 2). Moreover, we found that *L. chinensis* individual sizes and phenotypic traits were negatively correlated with N, P, and C concentrations (*p *<* *.05, Figure 3a and Table S2).

### 3.3 Mowing drive the decrease of nutrient accumulations

**Figure 4:**
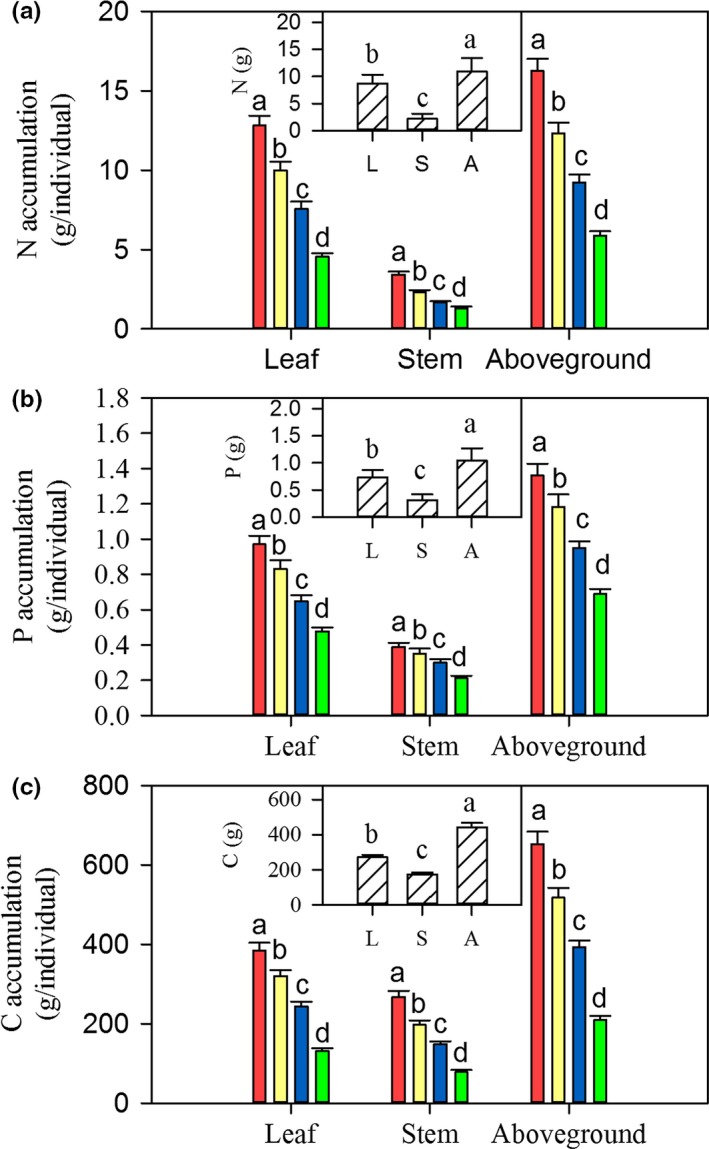
Effects of long‐term mowing frequencies (large figures) and sampling position (small figures) on N (a), P (b), and C (c) accumulation in *Leymus chinensis* individuals. Annotations and abbreviations as in Figures 1 and 2

With increasing mowing frequencies, the accumulated amounts of N, P, and C gradually and significantly decreased (*p *<* *.05, Figure 4). At the organ scale, nutrient accumulation amounts in *L. chinensis* leaves were significantly higher than in stems (*p *<* *.05, Figure 4). We also detected a positive correlation between *L. chinensis* individual size and accumulated amounts of N, P, and C (*p *<* *.05, Figure 3b and Table S3). Moreover, the concentrations were negatively correlated with the accumulation of N, P, and C in the biomass of leaves, stems, and the whole aboveground (Figure S2). In general, the most significant trade‐off was found for P between the two nutrient strategies of the three elements (*p *<* *.01, Figure S2).

### 3.4 Relations between ecological stoichiometry and plant size

**Figure 5:**
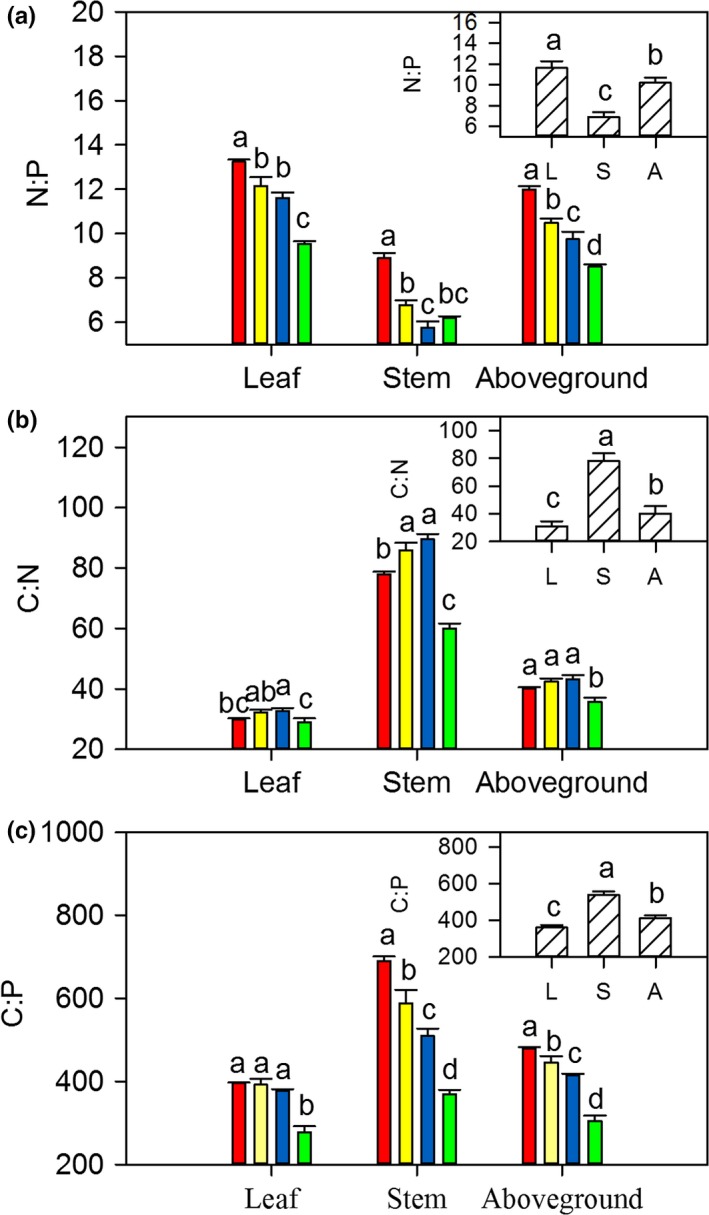
Effects of long‐term mowing frequencies (large figures) and sampling position (small figures) on ecological stoichiometry of C:N:P in *Leymus chinensis* individuals. Annotations and abbreviations as in Figures 1 and 2

Ecological stoichiometry showed that N:P ratios significantly decreased with increasing mowing frequencies (*p *<* *.05). The N:P ratios in leaves were significantly higher than those in stems (*p *<* *.05, Figure 5a). The C:N ratios in leaves and stems first increased and then decreased with increasing mowing frequencies (Figure 5b). In contrast, C:P ratios were negatively correlated with mowing frequencies (*p *<* *.05, Figure 5c). Moreover, C:N and C:P ratios were significantly lower in leaves than in stems (*p *<* *.05, Figure 5). In addition, N:P ratios were linearly correlated with mowing intensity (*p *<* *.05). Ratios of C:N and C:P of the whole *L. chinensis* individual were positively correlated with plant size, following an exponential pattern (*p *<* *.01, Figure 3c and Table S4).

### 3.5 Effects of mowing frequencies on nutrient allocation

**Figure 6:**
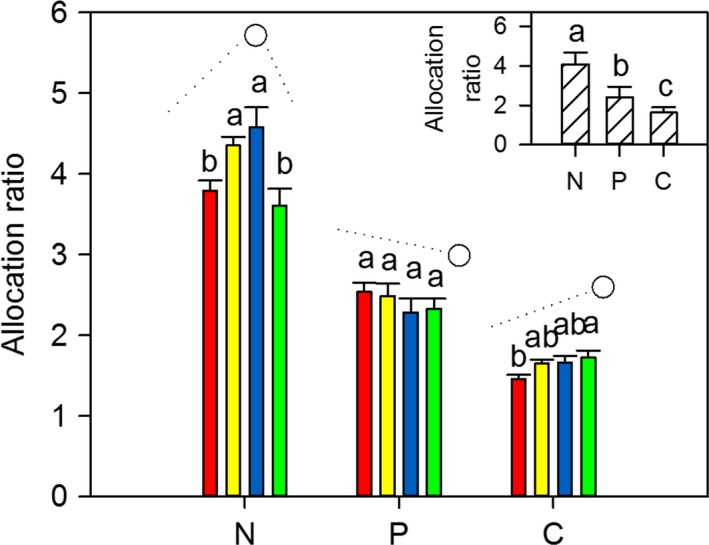
Effects of long‐term mowing frequencies (large figures) on N, P, C allocations in leaves and stems of *Leymus chinensis* individuals. The small figure indicates the comparison N, P, C allocations ratios. Annotations as in Figures 1 and 2

**Figure 7:**
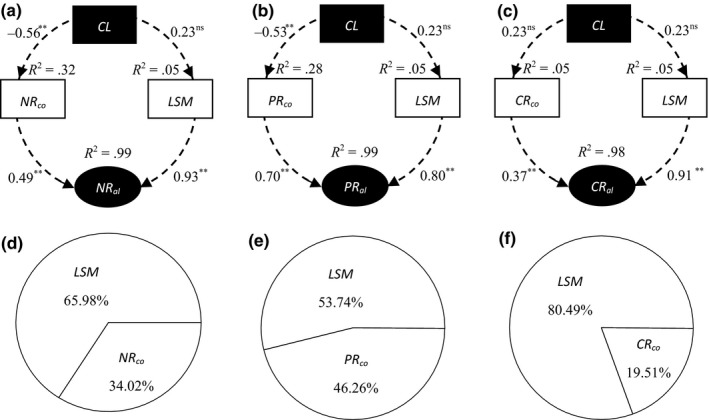
Final results of the structure equation modeling analysis of the effects of mowing frequencies on N (a), P (b), and C (c) allocation in *Leymus chinensis* in semi‐arid grassland. The proportions of plant N (d), P (e), and C (f) allocation variation in *L. chinensis* individuals are explained by the leaf to stem ratios of nutrient concentrations and biomass allocation. Values associated with arrows represent standardized path coefficients; *R* ^2^ values associated with response variables indicate the proportion of variation explained by relationships with other variables. **p < .05; ns, *p *> .05. Abbreviations: XR_co_, leaf to stem ratios of N, P, and C concentration; XR_al_, leaf to stem ratios of N, P, and C accumulation

We observed three different response patterns in the allocation ratios of N, P, and C from leaves to stems (Figure 6). Allocation ratios of N first decreased and then increased with increasing mowing intensity. Allocation ratios of P and C were negatively and positively correlated with mowing intensity (*p *<* *.05). The allocation ratios were as follows: N > P > C (*p *<* *.05, Figure 6). In addition, the plant phenotypic traits of *L. chinensis* were correlated with the allocation ratios of N, P, and C by the tendencies of parabola, positive, and negative, respectively (*p *<* *.05, Figure 3d and Table S5). The results of structure equation modeling and partial least‐squares regression showed that the allocation patterns from leaves to stems in N, P, and C were co‐regulated by the biomass allocation and nutrient concentration ratios of leaves to stems (Figure 7).

## 4 DISCUSSION

As a dominant species in eastern Eurasian temperate grasslands, *L. chinensis* is sensitive to habitat changes and various disturbances (Li, Liu, et al., 2015). In general, phenotypic plasticity is an important strategy by which plants adapt to heterogeneous habitats and effectively gain access to resources (Davidson, Jennions, & Nicotra, 2011; Via et al., 1995). Studying changes in plant phenotypic plasticity and nutrient strategies subject to mowing can provide insight into the mechanisms of grassland productivity formation, which might be important for enhancing ecosystem functions (Grant, Kreyling, Dienstbach, Beierkuhnlein, & Jentsch, 2014). In this research, we revealed the ecological processes of how long‐term mowing‐induced changes in concentrations, accumulations, ecological stoichiometry, and allocations of key elements mediated by the variations in plant size of *L. chinensis*.

Our findings show that the majority of *L. chinensis* phenotypic traits tend to be reduced in response to long‐term mowing, which is consistent with the results from previous studies in many mowing experiments in semi‐arid grasslands (Li, Wu, et al., 2015; Spasojevic & Suding, 2012). We also found that the PI values of leaf and stem phenotypic traits were significantly positively correlated with mowing intensity. Mowing in the Inner Mongolia grassland of China is a primary management strategy and also simulates grazing (Baoyin et al., 2014). Previous studies have reported that phenotypic plasticity is an important mechanism for grazing avoidance in plants which experienced long‐term grazing (Fu, Thompson, Willms, & Mackay, 2005; McKinney & Fowler, 1991). Therefore, the results of this study and of previous studies indicate that there are a number of similarities in the changes in functional traits in response to mowing and grazing. Our finding that leaves were more stable than stems in response to mowing implies that plants might invest more photosynthetic products to leaves in order to improve fitness. We also detected that leaf number increased slightly in response to light mowing disturbance and then decreased significantly with increased mowing frequency. This result suggests that there is a trade‐off between leaf number and leaf size at the early stage of perennial mowing or in the case of slight disturbance, implying that plant will produce a compensatory growth at individual scale (Lennartsson, Ramula, & Tuomi, 2018).

The contents of key nutrient elements in aboveground tissues are important for plant growth and development in nutrient‐poor ecosystems (Fay et al., 2015). In this study, the finding that heavy mowing significantly increases N, P, and C concentrations does not support our initial hypothesis that plant morphological plasticity in response to artificial mowing rooted in the decreasing nutrient concentrations in plant tissues. To date, there are no studies on the in‐depth mechanisms of the mowing‐induced increase in nutrient contents. Theoretically, this may be related to the three previously discussed possible reasons, that is, the growth dilution hypothesis, “functional equilibrium” theory and the nutrient competition hypothesis (Li, Liu, et al., 2016). Firstly, if the decrease in the accumulation of leaf biomass is more than the decrease in nutrient acquisition under mowing, nutrient concentrations will decrease (Bai et al., 2012; Shi et al., 2013). This is possibly the reason why we found significant trade‐offs between the concentrations and accumulation of N, P, and C in leaves, stems, and aboveground biomass. In addition, we speculate that this may also be associated with the changes in nutrient competition patterns. Secondly, the “functional equilibrium” theory predicated that plants respond to a decrease in aboveground resources with increased allocation to shoots (Poorter & Nagel, 2000). Therefore, mowing will promote more biomass to allocate to the leaf obtaining the most light resource (Chapin, Schulze, & Mooney, 1990). Thirdly, the competition of grassland plants for soil nutrients significantly weakened in mowed habitats, while in nonmowed habitats, increased nutrient concentrations were promoted (Lü et al., 2014; Veen, de Vries, Bakker, van der Putten, & Olff, 2014). These results are in agreement with a number of empirical studies on changes in soil nutrient availability and nutrient competition patterns with increased mowing disturbance (Helsen, Ceulemans, Stevens, & Honnay, 2014; Tilman & Wedin, 1991).

The stoichiometry of C:N:P in plant tissues is associated with plant growth strategies, which strongly influence its adaptation to various abiotic or abiotic disturbances, such as mowing (Hillebrand & Kahlert, 2001). Our results show that the N:P ratios in leaves and stems significantly decreased with increasing mowing frequencies. Previous studies have revealed that decreasing N:P ratios indicate N‐limitation in plant growth and development (Han et al., 2014). Hence, it is likely that heavy mowing limits *L. chinensis* growth through causing N deficiency, that is, increasing N requirement of the plant and decreasing N availability in the soil. Moreover, we detected that the N:P ratios in leaves were significantly higher than those in stems of *L. chinensis*. We speculate that this is most likely associated with the adaptive changes in allometry between leaves and stems, as shown previous studies (Li, Wu, et al., 2015). Because of the requirement of biosynthesis in protein‐associated compounds, plants developed the adaptive strategy of allocating more N to leaves than to stems.

To some extent, the ratios of C:P and C:N ratios in plant tissues can be a predictive indicator for nitrogen‐ and phosphorus‐use efficiency (Hidaka & Kitayama, 2013). We found that the C:N ratios in leaves and stems first increase and then decrease with increasing mowing frequencies, implying that moderate mowing is likely to increase the potential for N use efficiency. However, in our study, C:P ratios were negatively correlated with mowing frequencies, resulting in a lower P use efficiency in *L. chinensis* leaves and stems.

Our results demonstrated that the decrease in plant height after long‐term mowing corresponds with an increased C allocation from leaf to stem of *L. chinensis* at the individual scale, suggesting that an *L. chinensis* individual invests more of its photosynthetic products in leaves than in stems during plant miniaturization. In general, leaves are among the most important functional organs of a plant and are required for photosynthesis, transpiration, and nutrient use (Bloomfield, Farquhar, & Lloyd, 2014). Long‐term mowing, the removal of photosynthetic shoot tissue, limits plant photosynthesis (Thorne & Frank, 2009). It is possible that the mowing‐induced increase in the leaf to stem biomass ratio compensates the ability to carry out photosynthesis in the presence of defoliation each year. Potentially, increased leaf to stem biomass associated with individual plant miniaturization may be a mechanism for plant adaptation to mowing‐induced changes related to plant–soil interactions and ecosystem functioning (Sørensen, Kytöviita, Olofsson, & Mikola, 2008).

## 5 CONCLUSION

We conclude that mowing intensity is negatively correlated with phenotypic trait values and positively correlated with variability in plant functional traits in *L. chinensis* populations. In plants affected by mowing, the concentrations of N, P, and C in leaves and stems were significantly increased, negatively correlating with plant individual sizes. Moreover, the results show significant trade‐offs between the concentrations and accumulation of N, P, and C in leaves, stem, and aboveground biomass. The N:P ratios of *L. chinensis* aboveground biomass, linearly correlating with plant size, significantly decreased with increasing mowing frequencies. In contrast, the C:N and C:P ratios of *L. chinensis* individuals were positively correlated with plant size and showed an exponential pattern. With increasing mowing intensity, *L. chinensis* size was correlated with the allocation ratios of N, P, and C by the tendencies of parabola, positive, and negative, respectively. Structure equation modeling showed that the allocation patterns from leaves to stems in N, P, and C were co‐regulated by biomass allocation and nutrient concentration ratios of leaves to stems. Our results reveal a mechanism for long‐term mowing‐induced changes in concentration, accumulation, ecological stoichiometry, and allocation of key elements mediated by the variations in plant sizes of perennial rhizome grass.

## CONFLICT OF INTEREST

None declared.

## AUTHOR CONTRIBUTIONS

Z.Y.L., T.B., and X.L.L. designed the study; Z.Y.L., J.S., and X.L.L. analyzed the data; Z.Y.L. and H.M. carried out the experiment; all co‐authors contributed to writing and discussions.
