# Supplementary material for: Plant sizes mediate mowing‐induced changes in nutrient stoichiometry and allocation of a perennial grass in semi‐arid grassland
Source: Ecol Evol. 2018 Feb 16;8(6):3109–18. doi: 10.1002/ece3.3866 (PMC5869294; doi:10.1002/ece3.3866)
Supplement: Supplementary file 1 [file ECE3-8-3109-s001.docx]

**Supporting files**

**Figure S1.** Variance contribution of the five axes in the principal components analysis (PCA) for *Leymus chinensis* traits.

**Fig.S2.** Tradeoffs between concentrations and accumulation of N, P, C in leaf (a), stem (b), and aboveground biomass(c) of *Leymus chinensis* individuals. The tradeoffs were tested by correlation coefficients between concentrations and accumulation of N, P, C.

**Table S1.** Relationships of plant traits of *Leymus chinensis* individuals with the five principal components scores. Symbols: **, *P* < 0.01; *, *P* < 0.05, ns, *P* > 0.05.

| Plant trait | PCA 1 | PCA 2 | PCA 3 | PCA 4 | PCA 5 |
| --- | --- | --- | --- | --- | --- |
| Height | 0.96^**^ | 0.13^ns^ | 0.16^ns^ | 0.08^ns^ | -0.08^ns^ |
| Leaf number | 0.36^**^ | 0.91^**^ | -0.05^ns^ | -0.13^ns^ | 0.02^ns^ |
| Leaf length | 0.95^**^ | 0.07^ns^ | -0.02^ns^ | 0.11 | -0.14^ns^ |
| Leaf width | 0.88^**^ | -0.04^ns^ | 0.13^ns^ | 0.32^*^ | -0.09^ns^ |
| Leaf area | 0.94^**^ | 0.26^*^ | 0.00^ns^ | 0.20^ns^ | 0.05^ns^ |
| Specific leaf area | -0.18 | 0.01^ns^ | 0.08^ns^ | -0.03^ns^ | 0.98^**^ |
| Leaf biomass | 0.89^**^ | 0.32^*^ | -0.03^ns^ | 0.20^ns^ | -0.19^ns^ |
| Stem length | 0.37^**^ | -0.13^ns^ | 0.24 | 0.88^**^ | -0.04^ns^ |
| Stem diameter | 0.91^**^ | 0.19^ns^ | 0.28^*^ | 0.04^ns^ | -0.10^ns^ |
| Stem biomass | 0.85^**^ | 0.28^*^ | 0.29^*^ | 0.27^*^ | -0.15^ns^ |
| Aboveground biomass | 0.89^**^ | 0.31^*^ | 0.12^ns^ | 0.24^ns^ | -0.17^ns^ |
| Leaf to stem biomass ratio | -0.15^ns^ | 0.05^ns^ | -0.96^**^ | -0.18^ns^ | -0.09^ns^ |

**Table S2.** Correlations between various plant traits and tissue nutrient concentrations in leaves, stems, and aboveground biomass of *Leymus chinensis* individuals (n=60). *LM*, Leaf biomass; *LN*, Leaf number; *LL*, Leaf length; *LW*, Leaf width; *LA*, Leaf area; *SLA*, Specific leaf area; *SM*, Stem biomass; *SL*, Stem length; *SD*, Stem diameter; *AM*, Aboveground biomass; *LSM*, Leaf to stem biomass ratio; *PH*, Plant height; *NC*, nutrient concentrations. **, *P* < 0.01; *, *P* < 0.05; ns, *P* > 0.05.

| Category | Plant  traits | *NC* of leaf | | |  | *NC* of stem | | |  | *NC* of aboveground | | |
| --- | --- | --- | --- | --- | --- | --- | --- | --- | --- | --- | --- | --- |
|  |  | N | P | C |  | N | P | C |  | N | P | C |
| Leaf  traits | *LM* | -0.31^*^ | -0.72^**^ | -0.29^*^ |  | -0.57^**^ | -0.76^**^ | -0.03^ns^ |  | -0.40^**^ | -0.76^**^ | -0.29^*^ |
|  | *LN* | -0.24^ns^ | -0.51^**^ | -0.39^**^ |  | -0.54^**^ | -0.36^**^ | -0.01^ns^ |  | -0.29^*^ | -0.46^**^ | -0.36^**^ |
|  | *LL* | -0.33^*^ | -0.74^**^ | -0.23^ns^ |  | -0.57^**^ | -0.82^**^ | 0.07^ns^ |  | -0.42^**^ | -0.79^**^ | -0.20^ns^ |
|  | *LW* | -0.22^ns^ | -0.55^**^ | -0.12^ns^ |  | -0.35^**^ | -0.68^**^ | -0.10^ns^ |  | -0.33^**^ | -0.64^**^ | -0.20^ns^ |
|  | *LA* | -0.23 | -0.67^**^ | -0.27^*^ |  | -0.51^**^ | -0.77^**^ | -0.05^ns^ |  | -0.34^**^ | -0.73^**^ | -0.29^*^ |
|  | *SLA* | 0.42^**^ | 0.39^**^ | 0.01^ns^ |  | 0.34^**^ | 0.19^ns^ | -0.16^ns^ |  | 0.36^**^ | 0.32^*^ | -0.07^ns^ |
| Stem  traits | *SM* | -0.28^*^ | -0.68^**^ | -0.24^ns^ |  | -0.51^**^ | -0.74^**^ | -0.16^ns^ |  | -0.45^**^ | -0.75^**^ | -0.34^**^ |
|  | *SD* | 0.09 ^s^ | -0.14^ns^ | 0.27^*^ |  | 0.12^ns^ | -0.30^*^ | -0.17^ns^ |  | -0.03^ns^ | -0.24^ns^ | 0.13^ns^ |
|  | *SL* | -0.32^*^ | -0.75^**^ | -0.29^*^ |  | -0.60^**^ | -0.81^**^ | -0.13^ns^ |  | -0.48^**^ | -0.82^**^ | -0.37^**^ |
| Whole  plant  traits | *AM* | -0.30* | -0.71^**^ | -0.27^*^ |  | -0.56^**^ | -0.77^**^ | -0.09^ns^ |  | -0.43^**^ | -0.77^**^ | -0.32^*^ |
|  | *LSM* | 0.18^ns^ | 0.20^ns^ | -0.12^ns^ |  | 0.13^ns^ | 0.19^ns^ | 0.27^*^ |  | 0.42^**^ | 0.28^*^ | 0.10^ns^ |
|  | *PH* | -0.33^*^ | -0.76^**^ | -0.28^*^ |  | -0.60^**^ | -0.85^**^ | -0.02^ns^ |  | -0.47^**^ | -0.83^**^ | -0.30^*^ |

**Table S3.** Correlations between various plant traits and nutrient accumulation in leaves, stems, and aboveground biomass in *Leymus chinensis* individuals (n=60). *NA*, nutrient accumulation. Other abbreviations and symbols as in Table S2.

| Category | Plant  traits | *NA* of leaf | | |  | *NA* of stem | | |  | *NA* of aboveground | | |
| --- | --- | --- | --- | --- | --- | --- | --- | --- | --- | --- | --- | --- |
|  |  | N | P | C |  | N | P | C |  | N | P | C |
| Leaf  traits | *LM* | 0.98^**^ | 0.95^**^ | 0.99^**^ |  | 0.88^**^ | 0.79^**^ | 0.93^**^ |  | 0.97^**^ | 0.95^**^ | 0.98^**^ |
|  | *LN* | 0.56^**^ | 0.53^**^ | 0.57^**^ |  | 0.40^**^ | 0.54^**^ | 0.50^**^ |  | 0.54^**^ | 0.56^**^ | 0.55^**^ |
|  | *LL* | 0.89^**^ | 0.85^**^ | 0.92^**^ |  | 0.81^**^ | 0.66^**^ | 0.87^**^ |  | 0.89^**^ | 0.83^**^ | 0.91^**^ |
|  | *LW* | 0.85^**^ | 0.83^**^ | 0.86^**^ |  | 0.87^**^ | 0.72^**^ | 0.86^**^ |  | 0.87^**^ | 0.84^**^ | 0.88^**^ |
|  | *LA* | 0.96^**^ | 0.93^**^ | 0.96^**^ |  | 0.88^**^ | 0.75^**^ | 0.91^**^ |  | 0.96^**^ | 0.92^**^ | 0.96^**^ |
|  | *SLA* | -0.26^*^ | -0.29^*^ | -0.36^**^ |  | -0.21^ns^ | -0.32^*^ | -0.30^*^ |  | -0.25^ns^ | -0.31^*^ | -0.34^**^ |
| Stem  traits | *SM* | 0.91^**^ | 0.86^**^ | 0.92^**^ |  | 0.97^**^ | 0.86^**^ | 0.99^**^ |  | 0.94^**^ | 0.90^**^ | 0.97^**^ |
|  | *SD* | 0.49^**^ | 0.48^**^ | 0.48^**^ |  | 0.68^**^ | 0.50^**^ | 0.58^**^ |  | 0.54^**^ | 0.51^**^ | 0.53^**^ |
|  | *SL* | 0.87^**^ | 0.80^**^ | 0.88^**^ |  | 0.90^**^ | 0.76^**^ | 0.93^**^ |  | 0.89^**^ | 0.83^**^ | 0.92^**^ |
| Whole  plant  traits | *AM* | 0.96^**^ | 0.93^**^ | 0.98^**^ |  | 0.94^**^ | 0.84^**^ | 0.97^**^ |  | 0.97^**^ | 0.94^**^ | 0.99^**^ |
|  | *LSM* | -0.10^ns^ | -0.03^ns^ | -0.12^ns^ |  | -0.45^**^ | -0.44^**^ | -0.41^**^ |  | -0.18^ns^ | -0.17^ns^ | -0.25^ns^ |
|  | *PH* | 0.90^**^ | 0.84^**^ | 0.92^**^ |  | 0.88^**^ | 0.72^**^ | 0.93^**^ |  | 0.91^**^ | 0.84^**^ | 0.94^**^ |

**Table S4.** Correlations between various plant traits and C: N: P ecological stoichiometry in leaf, stem, and aboveground biomass of *Leymus chinensis* individuals (n=60). ES, ecological stoichiometry. Other abbreviations and symbols are as in table S3.

| Category | Plant  traits | *ES* of leaf | | |  | *ES* of stem | | |  | *ES* of aboveground | | |
| --- | --- | --- | --- | --- | --- | --- | --- | --- | --- | --- | --- | --- |
|  |  | N:P | C:P | C:N |  | N:P | C:P | C:N |  | N:P | C:P | C:N |
| Leaf  traits | *LM* | 0.74^**^ | 0.11^ns^ | 0.65^**^ |  | 0.54^**^ | 0.45^**^ | 0.72^**^ |  | 0.74^**^ | 0.26^*^ | 0.74^**^ |
|  | *LN* | 0.51^**^ | 0.02^ns^ | 0.42^**^ |  | -0.01^ns^ | 0.47^**^ | 0.27^*^ |  | 0.37^**^ | 0.17^ns^ | 0.40^**^ |
|  | *LL* | 0.75^**^ | 0.14^ns^ | 0.68^**^ |  | 0.62^**^ | 0.47^**^ | 0.80^**^ |  | 0.78^**^ | 0.29^*^ | 0.79^**^ |
|  | *LW* | 0.60^**^ | 0.09^ns^ | 0.52^**^ |  | 0.64^**^ | 0.23^ns^ | 0.67^**^ |  | 0.65^**^ | 0.21^ns^ | 0.65^**^ |
|  | *LA* | 0.74^**^ | 0.02^ns^ | 0.60^**^ |  | 0.61^**^ | 0.37^**^ | 0.73^**^ |  | 0.77^**^ | 0.18^ns^ | 0.72^**^ |
|  | *SLA* | -0.17^ns^ | -0.43^**^ | -0.40^**^ |  | 0.02^ns^ | -0.38^**^ | -0.20^ns^ |  | -0.11^ns^ | -0.42^**^ | -0.33^*^ |
| Stem  traits | *SM* | 0.72^**^ | 0.09^ns^ | 0.62^**^ |  | 0.57^**^ | 0.36^**^ | 0.69^**^ |  | 0.70^**^ | 0.30^*^ | 0.74^**^ |
|  | *SD* | 0.29^*^ | 0.00^ns^ | 0.23^ns^ |  | 0.52^**^ | -0.18^ns^ | 0.32^*^ |  | 0.37^**^ | 0.02^ns^ | 0.32^*^ |
|  | *SL* | 0.78^**^ | 0.10^ns^ | 0.67^**^ |  | 0.60^**^ | 0.44^**^ | 0.75^**^ |  | 0.76^**^ | 0.31^*^ | 0.79^**^ |
| Whole  plant  traits | *AM* | 0.74^**^ | 0.10^ns^ | 0.65^**^ |  | 0.57^**^ | 0.42^**^ | 0.72^**^ |  | 0.74^**^ | 0.28^*^ | 0.75^**^ |
|  | *LSM* | -0.15^ns^ | -0.18^ns^ | -0.22^ns^ |  | -0.17^ns^ | -0.03^ns^ | -0.13^ns^ |  | -0.07^ns^ | -0.34^**^ | -0.26^*^ |
|  | *PH* | 0.79^**^ | 0.11^ns^ | 0.69^**^ |  | 0.66^**^ | 0.46^**^ | 0.82^**^ |  | 0.80^**^ | 0.31^*^ | 0.82^**^ |

**Table S5.** Correlations between various plant traits and nutrient allocation ratios of *Leymus chinensis* individuals. NR, nutrient allocation ratios (n=60). Other abbreviations and symbols are as in Table S4.

| Category | Plant traits | *NR* of N | *NR* of P | *NR* of C |
| --- | --- | --- | --- | --- |
| Leaf  traits | *LM* | 0.17 | 0.17 | -0.20 |
|  | *LN* | 0.35^**^ | -0.03 | -0.06 |
|  | *LL* | 0.14 | 0.21 | -0.23 |
|  | *LW* | -0.14 | 0.08 | -0.34^**^ |
|  | *LA* | 0.11 | 0.18 | -0.25 |
|  | *SLA* | -0.16 | 0.00 | -0.09 |
| Stem  traits | *SM* | -0.14 | -0.08 | -0.47^**^ |
|  | *SD* | -0.45^**^ | -0.11 | -0.33^**^ |
|  | *SL* | -0.06 | -0.02 | -0.45^**^ |
| Whole  plant  traits | *AM* | 0.03 | 0.06 | -0.33^*^ |
|  | *LSM* | 0.88^**^ | 0.72^**^ | 0.92^**^ |
|  | *PH* | 0.02 | 0.11 | -0.38^**^ |
